# Serosorting Is Associated with a Decreased Risk of HIV Seroconversion in the EXPLORE Study Cohort

**DOI:** 10.1371/journal.pone.0012662

**Published:** 2010-09-09

**Authors:** Susan S. Philip, Xuesong Yu, Deborah Donnell, Eric Vittinghoff, Susan Buchbinder

**Affiliations:** 1 San Francisco Department of Public Health, San Francisco, California, United States of America; 2 Statistical Center for HIV/AIDS Research and Prevention, Seattle, Washington, United States of America; 3 University of California San Francisco, San Francisco, California, United States of America; Tulane University, United States of America

## Abstract

**Background:**

Seroadaptation strategies such as serosorting and seropositioning originated within communities of men who have sex with men (MSM), but there are limited data about their effectiveness in preventing HIV transmission when utilized by HIV-negative men.

**Methodology/Principal Findings:**

Data from the EXPLORE cohort of HIV-negative MSM who reported both seroconcordant and serodiscordant partners were used to evaluate serosorting and seropositioning. The association of serosorting and seropositioning with HIV seroconversion was evaluated in this cohort of high risk MSM from six U.S. cities. Serosorting was independently associated with a small decrease in risk of HIV seroconversion (OR = 0.88; 95%CI, 0.81–0.95), even among participants reporting ≥10 partners. Those who more consistently practiced serosorting were more likely to be white (p = 0.01), have completed college (p = <0.0002) and to have had 10 or more partners in the six months before the baseline visit (p = 0.01) but did not differ in age, reporting HIV-infected partners, or drug use. There was no evidence of a seroconversion effect with seropositioning (OR 1.02, 95%CI, 0.92–1.14).

**Significance:**

In high risk HIV uninfected MSM who report unprotected anal intercourse with both seroconcordant and serodiscordant partners, serosorting was associated with a modest decreased risk of HIV infection. To maximize any potential benefit, it will be important to increase accurate knowledge of HIV status, through increased testing frequency, improved test technology, and continued development of strategies to increase disclosure.

## Introduction

Over the past decade, reports of increases in unprotected anal intercourse among men who have sex with men (MSM) in multiple settings led to concern for subsequently rising rates of HIV and STDs which have been widely observed[1]. However, at least in a few areas, available epidemiologic data suggest that HIV incidence in MSM may not have increased as expected despite a rise in reported unprotected anal intercourse during the same period[2].

One possible explanation proposed for this discrepancy has been the observation that some MSM utilize certain HIV risk reduction strategies that are based on their own HIV infection status, and that of their partners. Various community-originated strategies have been reported from multiple areas[3], [4], [5] and have been called ‘seroadaptation’ and can include selectively choosing partners with an identical HIV serostatus to one's own, or choosing specific sexual practices based on a partner's serostatus. For example, one definition of the seroadaptation practice known as serosorting is that some MSM who practice anal intercourse use condoms with partners whose HIV infection status differs from theirs (serodiscordant partners) but have unprotected anal intercourse with seroconcordant partners[6]. Additionally, the terms strategic positioning or seropositioning have been interchangeably used to describe unprotected anal intercourse between HIV serodiscordant MSM, in which the HIV uninfected partner is preferentially insertive during anal intercourse [7].

However, practicing seroadaptation as a method of HIV prevention requires decision-making based on often-imperfect knowledge of HIV serostatus. Interviews with recently HIV infected gay men have reported failures of seroadaptation[8] and in a recent cross-sectional study of MSM attending a Seattle STD clinic, while seroadaptation practices were safer than unprotected anal intercourse without consideration of partner's infection status, they were associated with an increased risk of infection when compared to consistent condom use[9]. Finally, unprotected anal intercourse with partners believed to be HIV-uninfected has been associated with HIV seroconversion in cohort and case control studies of HIV-negative MSM[10], [11], [12]. Additional knowledge of whether and to what extent seroadaptation prevents infection in HIV-negative MSM is therefore important in determining whether these strategies should be recommended as effective HIV prevention tools for this group.

We evaluated the frequency and predictors of two seroadaptation strategies reported by HIV-negative MSM reporting multiple partners in the EXPLORE cohort: serosorting (defined in our study as preferential condom use during anal intercourse with HIV positive or unknown status partners rather than with HIV negative partners) and seropositioning (preference for the insertive rather than receptive role for anal intercourse with HIV positive or unknown status partners compared to HIV negative partners). Furthermore, we evaluated the association of these practices with HIV seroconversion.

## Methods

The EXPLORE study was a randomized trial of an individualized behavioral HIV prevention intervention in HIV-negative MSM in six U.S. cities from 1999–2003. The primary study outcome, HIV incidence, showed a modest decrease (HR = 0.82, p = 0.64–1.05) that did not reach statistical significance[13].

Full methods of the study have been described previously [14]. In brief, subject demographics were assessed at baseline, and risk behavior was ascertained using audio computer-assisted self-assessment at 6-month intervals for up to 48 months. HIV antibody testing was performed by enzyme linked immunosorbent assay at these semi-annual visits. From the sexual behavior data collected every six months, we assessed the total number of anal intercourse acts with HIV infected or unknown serostatus partners (HIV+/u) and HIV uninfected partners (HIV-), identified as with or without a condom, then further classified as insertive or receptive anal intercourse. Serosorting compared the occurrence of acts with versus without a condom in the HIV+/u compared to HIV- partners and was restricted to MSM reporting both serostatus partners and sex with and without a condom. Seropositioning compared the occurrence of insertive versus receptive acts in the HIV+/u versus HIV- partners, and was restricted to men reporting both serostatus partners and insertive and receptive sex. In all current analyses, we combined the contacts with HIV infected and HIV status unknown partners into a single response category (HIV+/u), both because of the relative infrequency of anal intercourse with HIV infected partners, and because public health messages advise that safest sex practices be used equally with HIV positive and unknown serostatus partners, given the strong association of unprotected anal intercourse with HIV status unknown partners with HIV seroconversion[10], [11], [12].

The baseline demographic characteristics and reported risk behaviors for the subgroups included in the seropositioning and serosorting analyses were each compared to the entire EXPLORE cohort.

For each participant, using all the episodes reported in a time period, a serosorting score was calculated as the odds of using a condom with HIV+/u partners, divided by the odds of using a condom with HIV- partners. Similarly, a seropositioning score was calculated as the odds of being the insertive partner in anal intercourse episodes with HIV+/u partners, divided by the odds of being the insertive partner in episodes with HIV- partners. For example, for a participant with a serosorting score of three, the odds of reporting condom use during anal intercourse with HIV+/u partners was three times higher than in episodes with their HIV- partners. In contrast, a participant with a seropositioning score of one was equally likely to be insertive during anal intercourse with HIV+/u and HIV- partners. Isolated zero cell values were replaced by 0.5 to avoid infinite values of the score; however, no score was computed if any marginal total of the two-by-two tabulation of contacts by partner serostatus and condom use or role was zero – for example, if no contacts with HIV+/u partners or no unprotected contacts were reported.

To identify correlates of serosorting, we compared the baseline characteristics of participants with scores four or more to those with scores of one or less, using t- or chi-square tests as appropriate. To a first approximation, these cutoffs result in comparing the upper and lower tertiles of the scores. For this analysis only, participant scores were based on anal intercourse contacts aggregated across all study visits.

To assess the associations of seropositioning and serosorting with HIV seroconversion, we used the same adaptation of the Cox model previously employed in the primary analyses of this outcome in EXPLORE[11]. Specifically, HIV seroconversion was assessed at each follow-up visit and treated as a discrete-time survival outcome. In these two models, log transformations of the participant seropositioning scores and serosorting scores were defined as time-dependent covariates, using only contacts reported from baseline through the current visit at which HIV seroconversion was being assessed. Log-transformation was used to achieve linearity and to avoid undue influence of outliers on the right of the distribution of scores. We adjusted for covariates previously used in EXPLORE analyses of HIV seroconversion, including race/ethnicity, numbers of sexual partners, self-report of sexually transmitted infections, methamphetamine and heavy alcohol use, depression, and use of drug and alcohol during sex. However, we did not adjust for more direct measures of sexual risk, in particular numbers of unprotected anal intercourse contacts by partner serostatus and role, since we saw these variables as direct mediators of seropositioning and serosorting.

Because prior EXPLORE analyses have found increased risk of seroconversion in association with higher numbers of partners perceived to be HIV-uninfected[11], the model for serosorting was also run stratified by number of partners. Similarly, the model effect of seropositioning was assessed within quartiles of reported condom use. In both these models, a Wald test for effect modification was performed.

## Results

Of the 4295 participants in EXPLORE, 4113 (96%) had at least one HIV test result during follow-up. Of these, 2623 (64%) reported anal intercourse with both HIV+/u and HIV- partners and inconsistent use of condoms and thus were eligible for inclusion in the serosorting analysis. In the group of men reporting anal intercourse with both HIV+/u and HIV- partners, 2667 (65%), reported assuming both receptive and insertive roles for anal intercourse and were included in the analysis of seropositioning. A total of 2345 men were eligible for inclusion in both analyses. Some EXPLORE participants reported other seroadaptation practices outside of those evaluated in our study, and so were not included in our analyses. For example, 670 (16%) reported zero sex acts with HIV+/u partners, 740 (18%) reported no unprotected sex, and 136 (3%) participants reported no anal intercourse. Table 1 shows the distribution of baseline demographic and risk behaviors in the complete EXPLORE cohort as well as the serosorting and seropositioning subcohorts.

**Table 1:** Demographic characteristics and risk behaviors reported at the baseline visit.

	EXPLORE (n = 4295)	%	Serosorting Cohort (n = 2623)	%	p-value†	Seropositioning Cohort (n = 2667)	%	p-value‡
Arm								
Intervention	2144	50	1253	48	0.0005	1288	48	0.01
Age								
<30	1727	40	1089	42	0.01	1116	42	0.0001
31–40	1665	39	1019	39		1045	39	
>40	903	21	515	20		506	19	
Race/ethnicity								
White	3112	73	1936	74	0.08	1983	74	0.0011
Black	281	7	161	6		153	6	
Hispanic	652	15	385	15		392	15	
Other	250	6	141	5		139	5	
Education								
Completed College	2757	64	1770	68	<0.0001	1790	67	<0.0001
In the past 6 months:								
10 or more male partners	1812	42	1283	49	<0.0001	1261	47	<0.0001
Reported HIV+ Partners	1215	28	809	31	<0.0001	814	31	<0.0001
Reported HIV unknown Partners	3354	78	2189	84	<0.0001	2204	83	<0.0001
Any Drug use	2977	69	1905	73	<0.0001	1938	73	<0.0001
Amphetamine use	552	13	388	15	<0.0001	385	14	0.0001
Injection Drug use	439	10	227	9	<0.0001	239	9	0.0006
Use of Alcohol or Drugs before sex	3088	72	1972	75	<0.0001	1994	75	<0.0001
In primary relationship	2074	48	1295	49	0.08	1318	49	0.06

† Serosorting Cohort compared to the entire EXPLORE Cohort.

‡ Seropositioning Cohort compared to the entire EXPLORE Cohort.

Compared to all EXPLORE participants, those in both subcohorts were slightly younger and more likely to be college educated and to have reported HIV risk behaviors including drug use and ten or more partners. There was also weak evidence that participants in the serosorting subcohort were more likely to be in a primary relationship, and that those in the seropositioning subcohort were more likely to be white. Assignment to the EXPLORE control arm was also associated with inclusion in both subcohorts.

**Figure 1:**
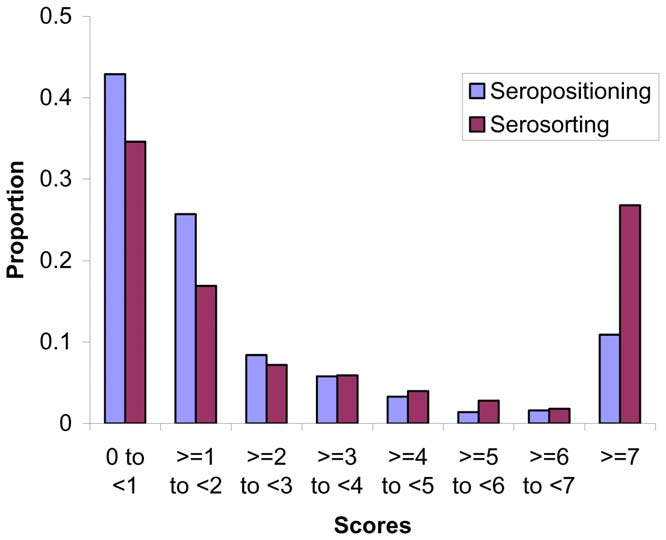
Distribution of seropositioning and serosorting scores.

The distribution of the serosorting and seropositioning scores is shown in Figure 1. Thirty five percent of the serosorting subcohort and 43% percent of the seropositioning subcohort had scores less than one. Serosorting was more consistently practiced than seropositioning: a score of two or higher was seen in 48% of participants in the serosorting subcohort, but in only 31% of those in the seropositioning subcohort. A score of four or greater was seen in 35% and 17% of the serosorting and seropositioning subcohorts, respectively.

As shown in Table 2, more consistent serosorters (those with a score of four or higher) were more likely than non-serosorters (those with a score of one or less) to be white, college-educated, and to be in a primary relationship. Consistent serosorters were less likely than non-serosorters to report 10 or more partners at baseline. There were no differences in EXPLORE study arm, age, HIV+/u partners, or drug use; although there was a trend toward decreased amphetamine use in the consistent serosorters, this did not achieve statistical significance.

**Table 2:** Comparison between serosorters (score> = 4) and non-serosorters (score< = 1) at baseline visit within the EXPLORE serosorting subcohort (n = 2623).

	Non-serosorters (n = 926)	%	Serosorters (n = 928)	%	p-values
Arm					0.85
Intervention	435	47	441	48	
Age					0.17
<30	381	41	412	44	
31–40	355	38	355	38	
>40	190	21	161	17	
Race/ethnicity					0.01
White	638	69	701	76	
Black	71	8	48	5	
Hispanic	162	17	132	14	
Other	55	6	47	5	
Education					
Completed College	572	62	650	70	0.0002
In the past 6 months:					
10 or more male partners	465	50	407	44	0.007
Reported HIV+ Partners	273	29	278	30	0.85
Reported HIV unknown Partners	756	82	759	82	0.98
Any Drug use	676	73	669	72	0.70
Amphetamine use	162	17	132	14	0.06
Injection Drug use	83	9	76	8	0.61
Use of Alcohol or Drugs before sex	688	74	703	76	0.5
In primary relationship	417	45	519	56	<0.0001

Results of the Cox proportional hazard model for the association of serosorting with HIV seroconversion are shown in Table 3. There were a total of 259 HIV seroconversions in EXPLORE, of which 175 (68%) were in the serosorting subgroup. Serosorting was independently associated with a 12% decreased risk of HIV seroconversion for each one unit increase in the natural log serosorting score (OR = 0.88; 95%CI, 0.81–0.95). The protective effect of serosorting did not differ by the number of reported partners in the stratified analysis, even for those reporting ten or more partners in the past six months (p = 0.59) (data not shown).

**Table 3:** Predictors of HIV seroconversion in the EXPLORE serosorting subcohort.

	Serosorting Subcohort			
	n at baseline (n = 2623)	Adjusted OR	95%CI	p-value
Serosorting		0.88	0.81, 0.95	<0.001
Race/ethnicity				
Non-black	2462	Reference		
Black	161	1.36	0.76, 2.45	0.30
No. of male sex partners				
0–3	484	Reference		
4∼9	855	1.53	0.92, 2.53	0.10
> = 10	1283	2.83	1.80, 4.43	<0.001
Amphetamines use	388	2.55	1.82, 3.59	<0.001
Heavy alcohol use	282	1.73	1.05, 2.83	0.03
Self-reported STDs				
No STDs	2379	Reference		
Gonorrhea	101	2.85	1.60, 5.07	<0.001
Chlamydia	140	1.76	0.86, 3.58	0.12
Syphilis	3	1.51	0.43,5.31	0.53
Depression scale				
7–12	1371	Reference		
13–17	875	1.73	1.22, 2.45	0.002
18–22	280	1.27	0.75, 2.14	0.38
23–28	87	2.24	1.21, 4.14	0.01
Use of alcohol or drugs before sex	1972	1.77	1.10, 2.83	0.02

Adjusted for race, number male partners, self-reported gonorrhea, depression, alcohol or drug use before sex, amphetamine use.

In contrast to serosorting, seropositioning was not significantly associated with HIV seroconversion in the adjusted model (OR = 1.02; 95%CI, 0.92–1.14), as shown in Table 4. In the analysis stratified by quartile of reported condom use (1–100%) no effect modification was detected (p = 0.63) (data not shown).

**Table 4:** Predictors of HIV seroconversion in the EXPLORE seropositioning subcohort.

		Seropositioning Subcohort		
	n at baseline (n = 2667)	Adjusted OR	95%CI	p-value
Seropositioning		1.02	0.92, 1.14	0.71
Race/ethnicity				
Non-black	2514	Reference		
Black	153	1.70	0.98, 2.94	0.06
No. of male sex partners				
0–3	513	Reference		
4∼9	891	1.62	0.99, 2.64	0.05
> = 10	1261	2.89	1.86, 4.51	<0.0001
Amphetamines use	385	2.43	1.73, 3.41	<0.0001
Heavy alcohol use	273	1.78	1.09, 2.92	0.02
Self-reported STDs				
No STDs	2424	Reference		
Gonorrhea	102	2.67	1.47, 4.85	0.001
Chlamydia	138	1.50	0.71, 3.18	0.29
Syphilis	3	2.28	0.75, 6.94	0.15
Depression scale				
7–12	1410	Reference		
13–17	871	1.82	1.29, 2.58	0.0007
18–22	285	1.47	0.89, 2.44	0.13
23–28	89	2.16	1.14, 4.10	0.02
Use of alcohol or drugs before sex	1994	1.68	1.07, 2.66	0.03

Adjusted for race, number male partners, self-reported gonorrhea, depression, alcohol or drug use before sex, amphetamine use.

## Discussion

Approximately 65% of the high risk EXPLORE cohort met our criteria for inclusion in either subcohort and among these, serosorting and seropositioning were both reported by a sizable minority. By definition, the seropositioning and serosorting cohorts comprise men who report both HIV-uninfected and HIV positive/unknown anal intercourse partners, as well as inconsistent condom use and/or role versatility, and are thus behaviorally at higher risk for HIV. For almost half of the serosorting subcohort, the odds of using a condom with HIV-infected or unknown status partners were at least twice that with partners believed to be HIV-uninfected.

Other studies in HIV-uninfected MSM in multiple areas including a Seattle STD clinic [9] a cross-sectional survey in Atlanta [15] and a cohort study in Australia[16] have defined serosorting as reporting unprotected anal intercourse only with partners believed to be HIV uninfected and found prevalences of this strategy of 26–38%. The prevalence of serosorting in our study is not directly comparable, because we focused on a subgroup of high-risk men who reported both HIV positive/unknown and HIV negative partners and both protected and unprotected anal intercourse. However, this did allow us to evaluate preferential condom use by partner type within a participant, which may identify those that intentionally utilized serososorting as a harm reduction strategy. We found that seropositioning was reported relatively less commonly than serosorting in EXPLORE, as was also seen in the Australian cohort[16]. However, nearly half of the participants in our analyses did not practice either serosorting or seropositioning, as shown by scores of ≤1.

To our knowledge, this is the first evaluation to utilize multivariable modeling to examine the effect of seroadaptation strategies on HIV incidence in a large prospective cohort of HIV-uninfected MSM whose reported behaviors place them among those at highest risk for HIV infection. Our finding that increasing serosorting was independently associated with a modest decrease in risk for HIV infection was encouraging, particularly since this protective effect was evident even among men with greater numbers of partners, which has been shown to be a independent risk factor for HIV infection in EXPLORE as well as another MSM cohort study[10], [11].

Furthermore, in previous studies, both serosorting and strategic positioning have been associated with an intermediate risk of HIV seroconversion when compared to no unprotected anal intercourse (lowest risk) and unprotected receptive anal intercourse with an HIV-infected partner (highest risk)[9], [16]. Certainly, seroconversion has also been reported despite serosorting in case control studies[12] and surveys of MSM[8]. Modeling studies have also suggested that attempts at serosorting could paradoxically increase risk for HIV infection, particularly in the setting of acute HIV, a stage of disease which is suspected to be highly infectious due to high viral loads, but during which conventional HIV antibody tests are negative[17].

In contrast to our findings, there are several reasons why we might have expected an increased risk of HIV infection associated with serosorting or seroadaptation in this study. For one, many men are unaware of their HIV infection status, and also may make assumptions about a partner's HIV status[15], [18], [19]. Additionally, as shown in a recent study of over 1800 HIV infected MSM, even for those who are aware of their own status, disclosure to sex partners can be complex and fraught with difficulty[20]. It is possible that the regular frequency of HIV testing, the enhanced participant-centered counseling that was provided to all EXPLORE participants or our inclusion criteria for these analyses contributed to our findings.

In addition to issues of generalizabilty, the study did have some additional limitations. Assessments of seropositioning and serosorting relied on participant report, which could have been subject to desirability bias despite attempts to minimize this by utilizing computer assisted self-survey. We were also unable to directly assess participant intention to practice seropositioning and serosorting as risk reduction strategies, although our study design did allow for measurement of the magnitude of these behaviors; neither could we evaluate differences in reported behavior with regular versus casual partners. Our findings could be biased by unmeasured confounders, including time-dependent factors such as knowledge of partner status. In addition our findings cannot be extended to MSM who are monogamous, do not practice anal intercourse, or who have only HIV-negative or HIV positive or unknown status partners. Finally, we measured only two specific seroadaptation behaviors out of the many that exist, including some which were reported by EXPLORE participants, and so cannot speak to the efficacy of those other practices.

Despite concerns that serosorting may contribute to unacceptable risk of HIV infection in MSM[21], our findings were that it did not increase risk and was even associated with a small protective effect. However, given this small magnitude of effect, counseling and health messages should continue to emphasize condom use and reducing numbers of partners as the mainstays of individual prevention efforts. Our findings, along with other studies, suggest that serosorting may have a role as a harm reduction strategy for MSM who currently practice unprotected anal intercourse with partners without serosorting. However there is currently no evidence to suggest that serosorting is as safe as consistent condom use and limiting numbers of partners.

Further qualitative studies could evaluate intention and skills associated with MSM who practice these behaviors, and also whether there are potentially modifiable factors that could be used to encourage and support serosorting as a harm reduction approach for MSM who do not always use condoms.

Although using condoms consistently and limiting numbers of partners remain central to HIV prevention, those MSM who choose serosorting as harm reduction should also be supported in this currently practiced, community-originated strategy. First, since disclosure is the keystone of any seroadaptation strategy, efforts to decrease barriers and routinize frequent HIV testing among MSM must be a continued priority of public health. However, since frequent testing alone may be insufficient due to limitations of antibody testing during acute HIV infection and the increased risk of HIV transmission associated with this period[22], improved tests, including pooled RNA testing and more sensitive fourth generation EIA, should become widely available to improve diagnostic accuracy of HIV testing for those at high risk of infection including MSM. Furthermore, additional research into interventions that encourage and support disclosure to partners is also necessary to maximize any potential HIV prevention benefit of serosorting as a harm reduction strategy.
